# Dissolvable microneedles loaded ginsenoside Rg3 liposome: a transdermal delivery approach for alopecia treatment

**DOI:** 10.1093/rb/rbae086

**Published:** 2024-07-16

**Authors:** Qin Yang, Peng Guo, Pengkun Lei, Qiaolin Yang, Yuchun Liu, Ya Tian, Wen Shi, Chunxiao Zhu, Min Lei, Rui Zeng, Chen Zhang, Yan Qu

**Affiliations:** State Key Laboratory of Southwestern Chinese Medicine Resources, School of Pharmacy, Chengdu University of Traditional Chinese Medicine, Chengdu 611137, China; State Key Laboratory of Southwestern Chinese Medicine Resources, School of Pharmacy, Chengdu University of Traditional Chinese Medicine, Chengdu 611137, China; State Key Laboratory of Southwestern Chinese Medicine Resources, School of Pharmacy, Chengdu University of Traditional Chinese Medicine, Chengdu 611137, China; State Key Laboratory of Southwestern Chinese Medicine Resources, School of Pharmacy, Chengdu University of Traditional Chinese Medicine, Chengdu 611137, China; State Key Laboratory of Southwestern Chinese Medicine Resources, School of Pharmacy, Chengdu University of Traditional Chinese Medicine, Chengdu 611137, China; State Key Laboratory of Southwestern Chinese Medicine Resources, School of Pharmacy, Chengdu University of Traditional Chinese Medicine, Chengdu 611137, China; State Key Laboratory of Southwestern Chinese Medicine Resources, School of Pharmacy, Chengdu University of Traditional Chinese Medicine, Chengdu 611137, China; State Key Laboratory of Southwestern Chinese Medicine Resources, School of Pharmacy, Chengdu University of Traditional Chinese Medicine, Chengdu 611137, China; Lu Huo Snow area E Se Limited Liability Company, Chengdu 626500, China; College of Pharmacy, Southwest Minzu University, Chengdu 610041, China; Key Laboratory of Research and Application of Ethnic Medicine Processing and Preparation on the Qinghai Tibet Plateau, Chengdu 610041, China; State Key Laboratory of Southwestern Chinese Medicine Resources, School of Pharmacy, Chengdu University of Traditional Chinese Medicine, Chengdu 611137, China; State Key Laboratory of Southwestern Chinese Medicine Resources, School of Pharmacy, Chengdu University of Traditional Chinese Medicine, Chengdu 611137, China

**Keywords:** cholesterol-free liposomes, microneedles, transdermal administration, Wnt/β-catenin pathway, alopecia treatment

## Abstract

The skin stratum corneum (SC) barrier function will interfere with the absorption of topical treatment and reduce the drug's therapeutic effect on alopecia. Microneedles (MNs) can penetrate the skin barrier and deliver drugs to the dermis. Furthermore, MNs can mechanically stimulate the skin, which promotes hair growth. Thus, we designed a green and dissolvable composite microneedle made of hyaluronic acid (HA) and *Bletilla striata* polysaccharide (BSP) to encapsulate cholesterol-free ginsenoside Rg3 liposomes (Rg3-LPs) to avoid cholesterol metabolism-producing testosterone to inhibit hair regeneration and minimize the effect of the SC barrier on liposomes absorption. HA and BSP can enhance the mechanical strength of Rg3-MNs to ensure the transport of liposomes to the hair follicle (HF) region while causing minimal skin irritation and guaranteeing cell compatibility. In addition, HA increased hair density and was more conducive to hair regeneration. In telogen effluvium (TE) and testosterone-induced androgenetic alopecia (AGA) animals, Rg3-MNs achieved comparable efficacy to minoxidil with low-frequency treatment and the quality of regenerated hair was higher. Furthermore, quantitative characterization and transcriptome sequencing results showed that Rg3-MNs promoted hair regeneration by promoting the expression of Wnt3a and Wnt10b genes, activating the Wnt/β-catenin pathway. Therefore, Rg3-MNs present broad prospects in the treatment of alopecia.

## Graphical abstract

**Figure:**
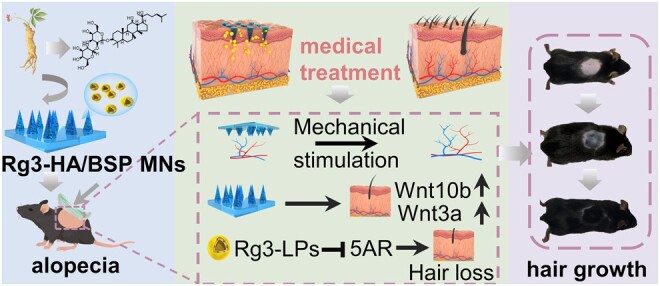

## Introduction

Alopecia affects an increasing number of people each year, with a younger age distribution, due to inheritance, environmental pollution, oxidative stress, microcirculation abnormalities, hair follicle (HF) degeneration, hormone disorders and other factors [1]. Clinically, non-scarring particularly telogen effluvium (TE), androgenetic alopecia (AGA) and alopecia areata (AA) are more prevalent [2]. Although hair loss does not directly cause physical pain like other diseases, if things continue in this manner, it will negatively impact the patient's social connections and self-esteem, which may result in depression and make them more susceptible to mental illnesses [3, 4]. The Food and Drug Administration (FDA) has approved for the utilization of finasteride and minoxidil to treat hair loss. However, because of the skin stratum corneum (SC) barrier, minoxidil absorption is limited, and long-term treatment might result in dermatitis and headaches, whilst finasteride can impair male fertility and cause depression. As a result, the patient's compliance is low, limiting the drug's overall efficacy [5, 6]. With hair loss gradually becoming a problem for all ages, it is significant to find low-frequency administration, safe and effective dosage forms and drugs as an alternative to hair loss treatment [7].

As a result of their nanoscale size and the benefits of system interaction with skin, nanocarriers like liposomes and nanoemulsions have been demonstrated to have potential for hair therapy [8]. One of them, the liposome injectable Doxil, received FDA approval in 1995. The safety and effectiveness of liposomes can be guaranteed [9]. Furthermore, liposomes can destroy the SC and effectively aim at skin layers while extending the duration of drug release [10]. Moreover, liposomes can improve medication stability and solubility, and improve insoluble drugs' bioavailability and biocompatibility [11]. Based on the advantages of liposomes, which are often used in transdermal delivery systems [12]. Cholesterol, a major film-forming component in conventional liposomes, inhibits the oxidation of phospholipids. Additionally, it can control the phospholipid membrane's fluidity, keep the membrane flexible and prevent drug leakage [13]. However, high cholesterol might result in improper liver and gallbladder metabolism and even atherosclerosis [14]. Additionally, cholesterol regulates the proliferation of stromal keratinocytes within HFs, oversees the formation of hair shafts and modulates the Wnt-β-catenin and hedgehog pathways [15]. The primary steroid hormone produced by cholesterol is testosterone, and the testosterone metabolite dihydrotestosterone (DHT) acts on the HFs, shrinking them and prolonging their telogen, which causes AGA [16]. As a consequence, prolonged cholesterol use will exacerbate baldness in individuals. Designing cholesterol-free liposomes is therefore essential to treating alopecia.

Ginsenoside Rg3 as an analog of cholesterol, the hydroxyl group at the C3 site can bind to the hydrophilic group of phospholipids, shorten the distance between phospholipid bilayers, decrease liposome particle size and boost liposome stability. It is advantageous to decrease the number of drugs and increase the duration of liposome blood circulation [17, 18]. It is worth noting that Rg3 can stimulate HFs and up-regulate the expression of vascular endothelial growth factor (VEGF) to stimulate hair development [19]. In addition, Rg3 inhibits 5a-reductase (5AR) and is regulated by ERK and AKT signaling pathways, which suppress DHT-triggered overexpression of androgen receptors, enhancing hair growth [20]. Thus, Rg3 is sufficient to replace cholesterol as a material for preparing liposomes for hair regeneration.

Topical liposome administration is safer than oral administration, but there are still limitations such as poor skin permeability and retention issues [21]. Microneedles (MNs) have micron-sized needle-like structures. Regardless of their molecular weight, they can penetrate the SC to surmount skin barrier and promote medication delivery to the epidermis and dermis. In recent times, they have frequently been employed for drug or vaccine delivery [22–24]. Through mechanical stimulation of the skin, MNs can also activate HF stem cells, up-regulate VEGF and stimulate the enhanced expression of Wnt/β-catenin pathway proteins pertinent to hair growth [25]. Furthermore, MNs can improve hair density and quality, either in conjunction with additional treatments or serving as a mechanism for medication administration [26]. Dissolvable MNs have the advantages of both appealing MNs and are more convenient and safer to use because of good biocompatibility and degradability [27].

**Scheme 1.**
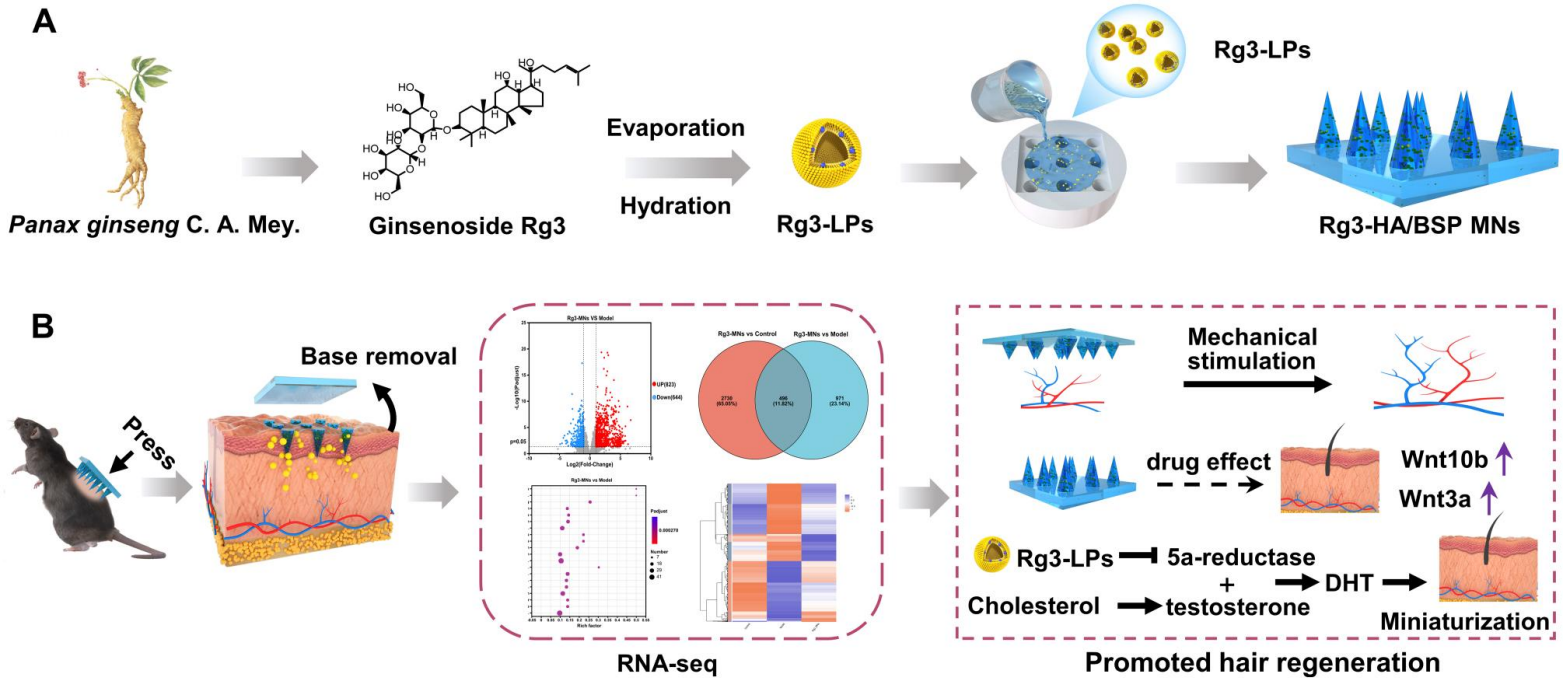
Schematic diagram of ginsenoside Rg3 microneedles patch for the treatment of alopecia. (**A**) To avoid the detrimental impact of cholesterol on hair regeneration, cholesterol-free Rg3 liposomes were loaded into soluble microneedles made of HA and BSP. Rg3-MNs dissolvable microneedles had no waste residue, and HA/BSP had good biocompatibility and degradability, which can enhance the mechanical strength of Rg3-MNs to ensure that Rg3-LPs are transported to the HFs region, which can exert long-term efficacy, cause minimal skin irritation and ensure biocompatibility. In addition, HA increased hair density and was more conducive to hair regeneration. (**B**) Rg3-MNs were employed to stimulate angiogenesis and activate the Wnt/β-catenin pathway, fostering HFs development and treating alopecia.

Consequently, encapsulating liposomes in dissolvable MNs and leveraging the channels produced by MNs to penetrate skin to transport medications and enhance drug utilization is an effective strategy to promote hair regeneration. In this study, we designed a green and dissolvable composite microneedle made of hyaluronic acid (HA) and *Bletilla striata* polysaccharide (BSP) to encapsulate cholesterol-free ginsenoside Rg3 liposomes (Rg3-LPs) to avoid cholesterol metabolism-producing testosterone to inhibit hair regeneration and minimize the effect of the SC barrier on liposomes absorption (Scheme 1). HA and BSP can enhance the mechanical strength of Rg3-MNs to transport liposomes to the HF region while causing minimal skin irritation and guaranteeing cell compatibility. In addition, HA increased hair density and was more conducive to hair regeneration. Then, the drug loading capacity, mechanical properties, safety performance, biodistribution of Rg3-MNs and drug release of Rg3-MNs were systematically evaluated. After that, Rg3-MNs were used to treat the established TE and testosterone-induced AGA models. Rg3-MNs were administered at a low frequency compared to frequently applying minoxidil to achieve similar efficacy. It could see hair regeneration in hair loss models, and the regenerated hair had a greater quality. Quantitative characterization and transcriptome sequencing results revealed that Rg3-MNs enhanced hair growth by upregulating Wnt3a and Wnt10b gene expression, thereby activating the Wnt/β-catenin pathway for hair development. Consequently, Rg3-MNs exhibited promising usage potential in the realm of treating alopecia.

## Materials and methods

### Materials and animals

Ginsenoside 20(S)-Rg3 was purchased from Chengdu DeSiTe Biological Technology Co., Ltd. (Chengdu, China). Cholesterol was supplied by Zhengzhou Feiman Biotechnology Co., Ltd. (Zhengzhou, China, mall.shiyanjia.com). Egg yolk phospholipid (EPC) was obtained from AVT (Shanghai) Pharmaceutical Tech Co., Ltd. Hyaluronate (HA, MW= 80–150 kDa) and minoxidil were supplied by Macklin. Polyvinyl alcohol (PVA) and methylene blue (MB) were provided by Chengdu Cologne Chemical Reagent Company (Chengdu, China). IR 780 was purchased from Sigma (USA). Testosterone and fluorescein isothiocyanate (FITC) were acquired from Shanghai Aladdin Biochemical Technology Co., Ltd. (Shanghai, China). C57BL/6 male mice (18–20 g, 7 weeks old) were obtained from SPF Biotechnology Co., Ltd. (Beijing, China).

### Preparation of Rg3-HA/BPS MNs

#### Preparation of BSP

BSP was created in the lab following the instructions of the manufacturer [28–30]. *Bletilla striata* tubers were into a powder sieve. Subsequently, they were extracted with 95% ethanol, petroleum ether and deionized water. The crude polysaccharide was obtained with 1/3 Sevage and 95% ethanol.

#### Preparation of Rg3-LPs

**Figure 1.**
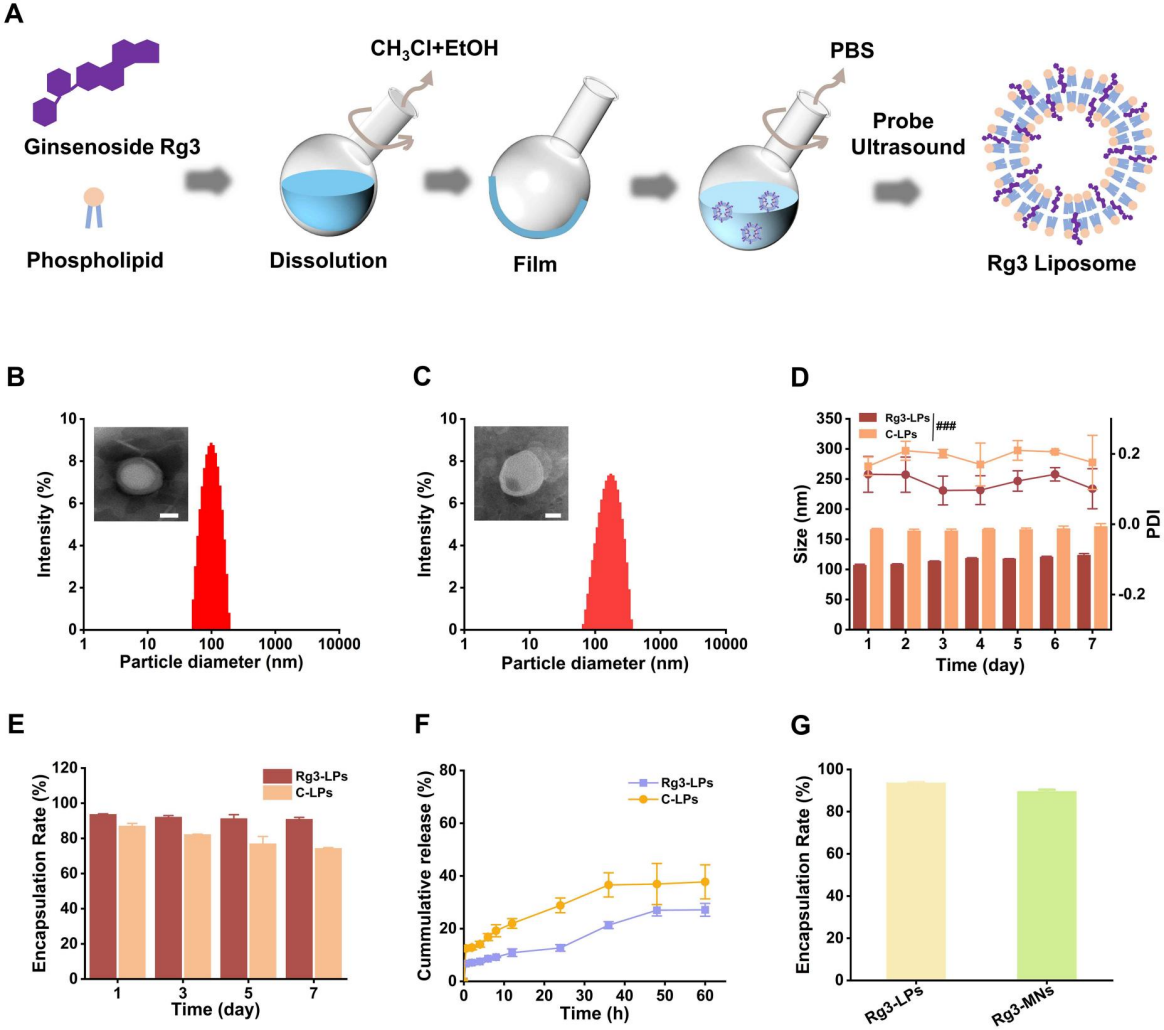
Schematic diagram of the preparation and characterization of liposomes. (**A**) Preparation of Rg3-LPs by film dispersion method. (**B**) TEM image and size distribution of Rg3-LPs. Scale bar: 50 μm (**C**) TEM image and size distribution of C-LPs. Scale bar: 50 μm (**D**) Particle size and PDI of Rg3-LPs and C-LPs within 7 days. (**E**) EE of Rg3-LPs and C-LPs within 7 days. (**F**) *In vitro* drug release. (**G**) EE of Rg3-LPs and Rg3-MNs. Data are presented as mean ± SD (*n* = 3). ^###^*P* < 0.001 vs the Rg3-LPs group.

Rg3-LPs were formulated utilizing the thin-film hydration technique (Figure 1A) [18]. Firstly, Rg3 and EPC (6:20, w/w) were accurately weighed, and chloroform and anhydrous ethanol (1:1, v/v) were added to stir and dissolve. Following rotary evaporation, hydration and sonication, Rg3-LPs were harvested. Then, Rg3, cholesterol and EPC (6: 6: 20, w/w/w) were prepared according to the above method, and Rg3 liposomes containing cholesterol (C-LPs) were obtained.

#### Preparation of Rg3-MNs

**Figure 2.**
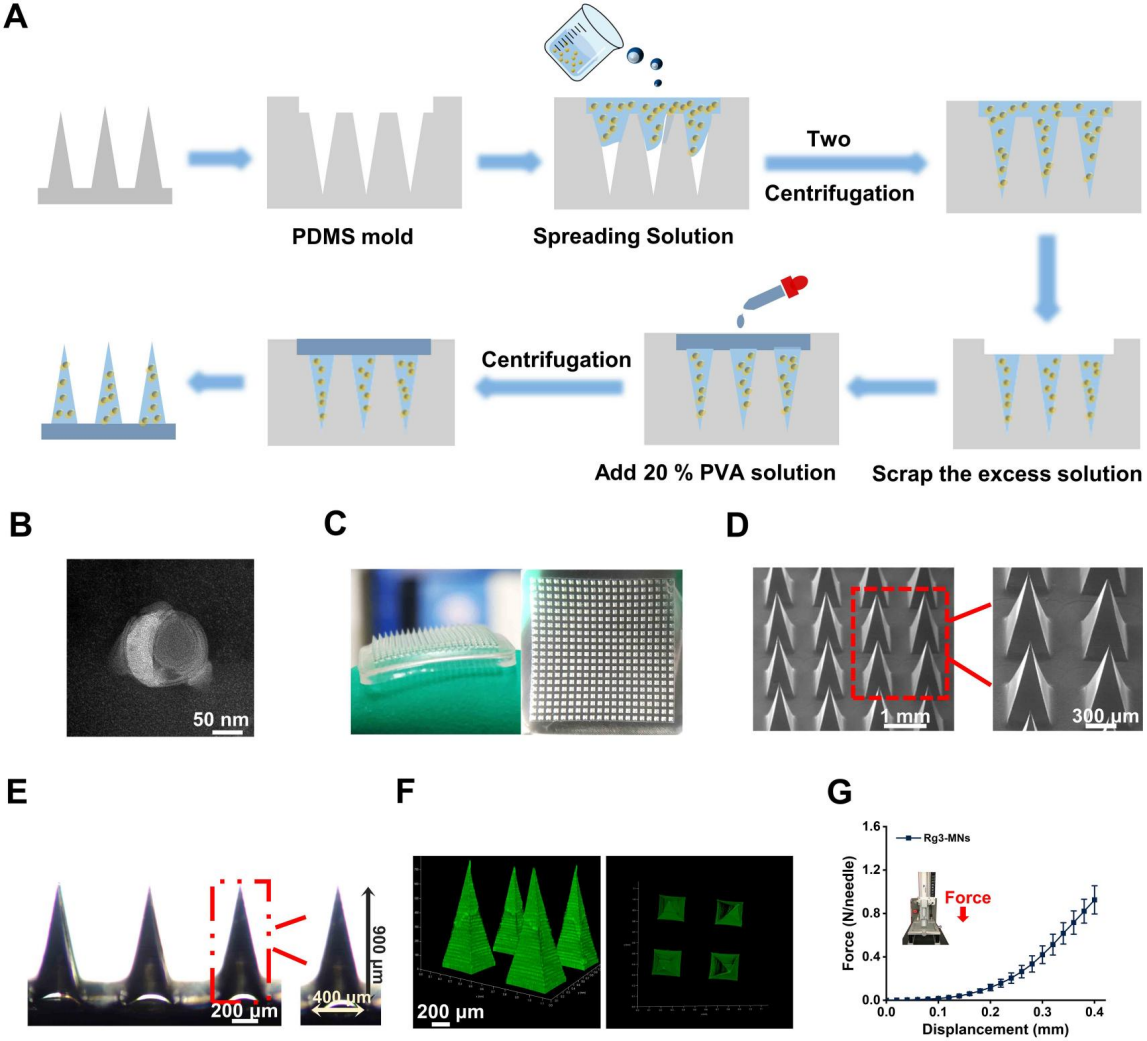
Schematic diagram of the preparation and characterization of Rg3-MNs. (**A**) Preparation of Rg3-MNs by two-step centrifugal method. (**B**) TEM image of redissolved Rg3-MNs. (**C**) Photograph of Rg3-MNs patch. (**D**) SEM image. (**E**) Optical microscope image. (**F**) 3D reconstructed image. (**G**) Force–distance curve. Data are presented as mean ± SD (*n* = 3).

The Rg3-HA/BSP-MNs were fabricated through two-step centrifugation (Figure 2A) [31]. HA and BSP (5:2, w/w) were added to Rg3-LPs solution to dissolve. The selection of these two polymers for microneedle production aims to achieve needles with optimal biocompatibility and dissolution rate. Furthermore, the composite MNs are mechanically strong, making them particularly suitable for drug delivery [32, 33]. After centrifuging, 1 ml of PVA solution (20%, w/v) was centrifuged to become supporting substrate. After drying can obtain Rg3-MNs. Similarly, FITC, MB, IR780 and DiR-labeled MNs were prepared.

### Characterization of Rg3-LPs

#### Physicochemical properties

The morphological features of Rg3-LPs were examined under a transmission electron microscope (TEM, Spectra 300 TEM, Thermo Fisher). Dynamic light scattering (Malvern Instruments, Malvern) determined the mean size and polydispersity index (PDI) of Rg3-LPs within 7 days. The encapsulation efficiency (EE) of Rg3 was determined based on the calibration curve (*y* = 0.1017*x* − 0.1645, *R*^2^ = 0.9995).

#### Rg3 release from liposomes

The dialysis method was employed to assess the *in vitro* release profile of C-LPs and Rg3-LPs [34]. Rg3-LPs and C-LPs solution were placed in a dialysis bag (molecular weight cut-off 8–14 kDa). Subsequently, they were enclosed in phosphate buffer (pH = 7.4) containing 5% Tween 80, respectively. About 0.6 ml of dialysate was taken out at a specific time point, methanol (1:1, v/v) was to dilute the demulsification and the same amount of isothermal phosphate buffer was added to the dialysis bag.

### Characterization of Rg3-MNs

#### Physical stability and content test

The Rg3-MNs were redissolved with 1 ml PBS, and the morphology, particle size and PDI of Rg3-LPs were used as indicators of physical stability. First, the Rg3-LPs suspension was diluted with 5 ml PBS and dried. After drying, the Rg3-LPs solution was negatively stained with 1% phosphorite acid. Finally, the morphology of Rg3-LPs was observed by the transmission electron microscopes.

Redissolved Rg3-MNs were diluted with methanol (1:5, v/v) to determine the content and EE of Rg3 in the MNs.

#### Morphology observation and mechanical strength test

The appearance of the MNs was evaluated using the scanning electron microscope (SEM) (SU9010, Hitachi) and the optical microscope (CX21, Olympic). The visualization of FITC-MNs was conducted utilizing by a confocal laser scanning microscope (CLSM; TCS SP8, Leica, Germany). The Texture Analyser (Shanghai Tengba Instrument Technology Co., Ltd.) was employed to assess the mechanical robustness of Rg3-MNs.

#### Insertion test

The Rg3-MNs were pasted onto a movable test probe with double-sided tape. Under the required 30 N force, the MNs were applied to the folded Parafilm M film.

To investigate whether Rg3-MNs could insert the skin, Rg3-MNs were tested on porcine skin and murine skin.

The excised porcine skin was penetrated with MB-Rg3-MNs to observe the insertion. Others, Rg3-MNs were implanted into the skin of hair removal C57BL/6 mice. The skin punctured by MNs was taken out and made into hematoxylin and eosin (H&E) staining sections. Lastly, the samples were examined under the optical microscope to further test the MNs insertion performance.

### Safety evaluation

#### TEWL measurement and skin irritation recovery test

Trans epidermal water loss (TEWL) measurements were taken before and following the insertion of Rg3-MNs, monitoring their return to baseline levels.

Rg3-MNs were inserted on the depilated mice and removed after pressing for 5 min. Then photographed before and at 0 and 60 min to observe whether there was erythema and micropore recovery.

#### Hemolytic experiment

Rg3-MNs (one piece, 130 μg), Blank-MNs (one piece) and 1 ml Rg3-LPs (130 μg/ml) were mixed with 8 ml normal saline and co-cultured in a biochemical incubator. Ear vein blood of New Zealand white rabbits was centrifuged to separate red blood cells and then washed with normal saline. Red blood cells (RBC) were added to normal saline to obtain 5% RBC suspension (v/v) [35].

The negative group and the positive group were saline, 0.1% Triton X-100 and RBC suspension (1:4, v/v) mixture, respectively. RBC suspension, Rg3-MNs and normal saline (2:1:7, v/v/v) was the experimental group; the mixture of Rg3-LPs and Blank-MNs with RBC suspension and normal saline (1:2:7, v/v/v) were prepared as the control group. The above solutions were cultured, after centrifugation, the absorbance was assayed by the microplate reader (SuPerMax3100, Flansh, Chain).

#### Biocompatibility *in vitro*

L929 cells were grown in DMEM containing 10% FBS and 1% antibiotics. Subsequently, they were plated in 96-well plates at a concentration of 5 × 10^3^ cells per well. The needle tips of Blank-MNs and Rg3-MNs were immersed in 10% FBS to obtain soaking solution of needle tips. After attachment, the DMEM was replaced with 0.2 mg/ml Blank-MNs, Rg3-MNs soaking solution and Rg3-LPs. CCK-8 Assay was measured *in vitro* cytotoxicity.

#### Dissolution experiment

Rg3-MNs were used on C57BL/6 mice and removed within the designated timeframe. The morphological changes of Rg3-MNs were inspected utilizing an optical microscope (DM750, Leica, Germany).

#### Biodistribution of Rg3-MNs

Rg3-MNs containing FITC were applied to the back skin of the C57BL/6 mice. After pressing, the patch base was removed after insertion into the skin. Then the image was observed under CLSM.

#### Transdermal delivery ability *in vitro*

The *in vitro* transdermal ability of Rg3-MNs was investigated by reference (Figure 4B) [36]. Rg3-MNs were applied to the isolated skin of C57BL/6 mice. The receiver pool was supplemented with PBS. At scheduled time points, receiver medium was taken out and fresh PBS was supplemented. The Rg3-LPs with the same content were used as the control group.

### Pharmacodynamic studies

#### *In vivo* treatment of TE model

To evaluate the effect of drug treatment, we established the TE model (Figure 5A) [37, 38]. The hair of 1 cm^2^ area was shaved and depilated with depilatory cream in seven-week-old C57BL/6 mice. The mice were randomly allocated into five distinct groups: control group, Rg3-LPs group, Blank-MNs group, Rg3-MNs group and minoxidil group. The minoxidil solution (3%, w/v) was formulated using a mixture of ethanol solution/1,2-propanediol/water (27:13:10, v/v/v). The minoxidil group received underwent topical administration of 3% minoxidil for seven consecutive days. For the Rg3-LPs, Blank-MNs and Rg3-MNs groups, mice were administered Rg3-LPs (1 ml, 130 μg/ml), Blank-MNs (one patch of drug-free MNs) and Rg3-MNs (one table, 130 μg) at a frequency of once every three days, concluding on the 7th day following depilation. The control group was without any treatment. On days 1, 5, 10 and 15 of the treatment periods, mice were photographed and their skin tone was assessed. On the 15th day after depilation, three mice with the best therapeutic effect in each group were selected for evaluation, and the diameter, density and skin thickness of the regenerated hair were observed. ImageJ software was utilized to calculate the coverage of regenerated hair.

#### Skin samples underwent histological analysis and immunofluorescence labeling

Animals were sacrificed on days 8 and 15 for H&E staining, then the H&E-stained sections were imaged under the microscope (3Dhistech Kft, Hungary) for histological analysis. On day 8 post-depilation, the samples were subjected to immunofluorescence staining, and the sliced skin samples were incubated with anti-VEGF (affinity) antibodies followed by the application of the corresponding secondary antibodies (ABclonal). DAPI (Beyotime) was employed for nuclear staining of cells. Immunohistochemical sections were visualized using the microscope (Soptop, Chain).

#### *In vivo* treatment of AGA model

Drawing from existing references, the AGA model was induced utilizing the testosterone solution (Figure 7A) [39–41]. Briefly, a 1 cm^2^ area on the dorsal skin of C57BL/6 male mice was depilated with animal clippers and hair-removing cream. The mice were randomly assigned to four distinct groups: model group, Blank-MNs group, Rg3-MNs group and minoxidil group. Testosterone (0.5%, w/v) was prepared in a mixture of ethanol solution/1,2-propanediol/water (27:13:10, v/v/v) and was applied twice daily to the shaved skin of all groups for 30 consecutive days to establish AGA model. The minoxidil group received topical application of 3% minoxidil for 13 consecutive days. In addition, mice in the Blank-MNs and Rg3-MNs groups were administered either Blank-MNs (one patch of drug-free MNs) or Rg3-MNs (one table, 130 μg) every three days, extending up to day 13 following depilation. Images were taken of the depilated area and skin color was observed. On day 30 post-depilation, three mice with the best therapeutic effect in each group were chosen for analysis, and regenerated hair's density, length and diameter were assessed. ImageJ software was used to quantify the coverage of regenerated hair.

#### Histological and immunofluorescence staining of skin samples

On day 14 after depilation, the dorsal skin samples underwent H&E staining, then H&E staining sections were histologically analyzed by microscope (3DHISTECH Kft, Hungary). The sliced skin samples were incubated with anti-VEGF (affinity) antibodies, anti-Ki67 (affinity) antibodies and anti-CD34 (affinity) antibodies, and then applied to the respective secondary antibodies (ABclonal). Nuclear staining was performed using DAPI (Beyotime) staining. Immunohistochemical sections were imaged under the microscope (Soptop, Chain). The staining area was quantified using the ImageJ software.

### Ethics statement

All animal experiments were approved by the Animal Ethics Committee of Chengdu University of Traditional Chinese Medicine (SYXK2020-124).

### Statistical analysis

Data were presented as mean ± standard deviation (SD). All statistical evaluations were performed using GraphPad Prism 8 software; ns, no statistical significance (*P* values > 0.05), ^#^*P* values < 0.05, ^##^*P* values < 0.01, ^###^*P* values < 0.001.

## Results

### Basic properties of BSP

For this experiment, we utilized BSP from the identical batch previously employed in our laboratory [42]. BSP comprises mannose and glucose at a ratio of 1.99:1, with (1 → 4)-linked-D-mannose serving as the primary link in the main chain. Purified BSP has Mw and Mn of 1.15 × 10^5^ g/mol and 1.12 × 10^5^ g/mol.

### Rg3-LPs characterization

The TEM images (Figure 1B and C) confirmed the successful preparation of Rg3-LPs and C-LPs. The Rg3-LPs were without cholesterol, the particle size was 106.88 ± 1.66 nm, the PDI was 0.142 ± 0.051. The particle size and PDI of C-LPs were 166.52 ± 1.40 nm and 0.165 ± 0.026. Furthermore, the EE of Rg3-LPs was 93.66% ± 0.34%. As illustrated in Figure 1D and E, the particle size of Rg3-LPs increased steadily within 7 days, and the PDI fluctuation was small, while the EE leakage rate was only 2.78%. However, the EE of C-LPs was 87.02% ± 1.47%, the particle size was relatively stable within 7 days and the PDI fluctuation was small, still, the EE leakage rate reached 12.66%, which confirmed that the prepared Rg3-LPs were concentrated and stable, the membrane stability of Rg3-LPs was better and the drug leakage rate was reduced. The higher EE and better stability are more favorable for the efficacy of MNs with limited drug loading.

From Figure 1F, we can observe that release of C-LPs reached 28.83% within 24 h, while Rg3-LPs was only 12.68%. In the next 36 h, the release of Rg3-LPs increased slowly to about 27.13%. The above datas show that the formulated Rg3-LPs exhibit an excellent and prolonged release effect, thereby minimizing the frequency of administrations and enhancing patient compliance.

### Physical stability and content of Rg3-LPs in MNs

TEM image (Figure 2B) displayed that Rg3-LPs were encapsulated in BSP and HA and maintained a complete spherical structure. After re-dissolving Rg3-MNs in PBS, the particle size was 109.44 ± 1.26 nm and the PDI was 0.246 ± 0.019. The content of Rg3 in the MNs was 127.13 ± 0.67 μg/patch and the EE was 89.55 ± 0.85 (Figure 1G), which was lower than the pre-loading value. This result may be due to the loss of liposomes after centrifugation and drying. However, the overall results indicate that Rg3-LPs have excellent stability in MNs, which is beneficial to exert efficacy.

### Rg3-MNs characterization

The images (Figure 2C) displayed that the MNs array was 400 (20 × 20). SEM and microscopy images (Figure 2D–F) implicated that the prepared microneedle base was about 400 μm wide and 900 μm high. The needle's tip was fashioned into a triangular cone-like shape, with strong drug loading force and a sharp tip, which was beneficial to puncture the skin.

In an effort to be more conducive to the delivery of drugs to the HF area and the treatment of hair loss, there are certain requirements for the mechanical properties of MNs. Upon analyzing the force–displacement curve depicted (Figure 2G), it is evident that the force intensifies proportionally with the displacement. Specifically, at a displacement of 0.34 mm, the mechanical force peaked at 0.62 ± 0.1 N/needle. Compared with previous studies [33], the Rg3-MNs developed in this study exhibited robust mechanical characteristics, enabling them to effectively penetrate the skin and deliver drugs in depth.

### Rg3-MNs insertion test *in vitro* and *in vivo*

**Figure 3.**
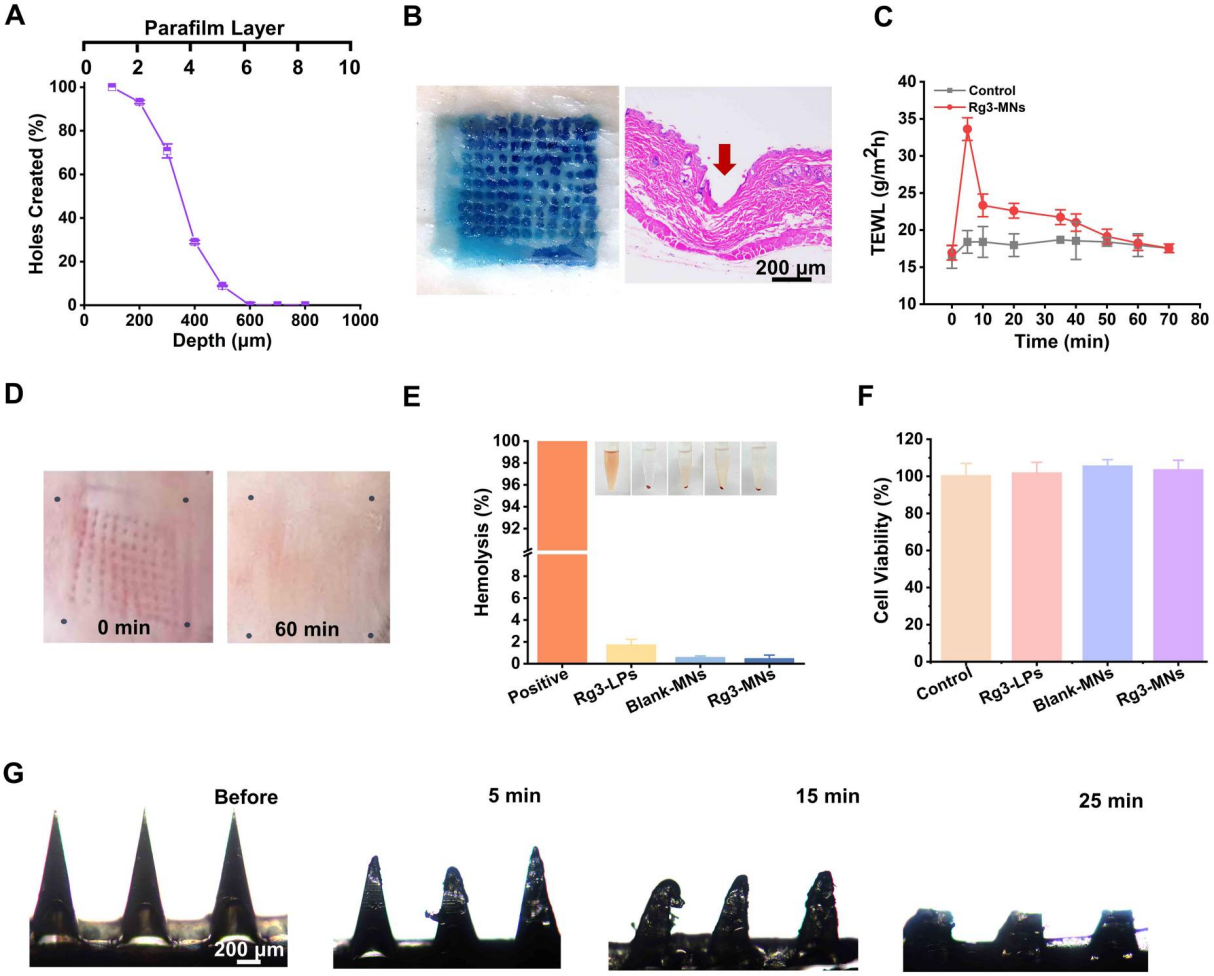
Characterization of Rg3-MNs. (**A**) The proportion of apertures formed and the penetration depth of MN. (**B**) Photograph of excised porcine skin and H&E staining sections. (**C**) Effect of MNs insertion on TEWL compared with the controls. (**D**) Image of mice skin at specified time points. (**E**)Hemolysis assay. (**F**) Cell viability assay (n = 6). Data are presented as mean ± SD (*n* = 3). (**G**) Dissolution of MNs at the different time points.

Each layer of Parafilm M exhibited a thickness of 100 μm, and the results (Figure 3A) reflected that the puncture depth of Rg3-MNs *in vitro* could reach 500 μm. The SC and epidermis possess a combined thickness of approximately 200 μm, and the thickness of the dermis is 1–3 mm. Parafilm M results confirm that Rg3-MNs have sufficient ability to puncture the SC, and epidermis and reach the dermis, which is convenient for drug delivery.

Figure 3B further demonstrates the *in vivo* and *in vitro* insertion performance of Rg3-MN. After the MB-MNs were inserted into the skin, most of the MNs could be inserted and left a clear pinhole mark. Additionally, skin histology showed that the insertion depth of Rg3-MN *in vivo* was about 330 μm. Although the insertion depth is lower than 500 μm, the skin thickness of the mice throughout the hair cycle is 300–700 μm, and the depth of pore formation can deliver the drug to the HF area [43].

### Safety evaluation results of MNs *in vivo*

#### Acute skin irritation test and TEWL measurement

TEWL value can reflect whether the skin barrier function is intact. As shown in Figure 3C, following Rg3-MNs treatment, there was a rapid surge in TEWL, which subsided to the original level within 70 min. It presented that this damage was reversible and could quickly return to normal levels, indicating that Rg3-MNs had good topical use safety.

After inserting the Rg3-MNs into the mice's skin for 5 mins, they were subsequently withdrawn, and the pinholes were clearly visible, but within 1 h, the pinhole imprints gradually disappeared (Figure 3D). In the stage of removing Rg3-MNs and skin self-recovery, without obvious erythema, swelling and other irritating reactions were observed in the skin. It is proved that the Rg3-MNs have good biocompatibility.

#### Hemolytic test

Figure 3E demonstrates that hemolysis occurred in all groups except the positive group, which proved the safety of Rg3-MNs, Rg3-LPs and Blank-MNs preparations.

#### *In vitro* cytotoxicity

By comparing the cell viability of Blank-MNs, Rg3-MNs and Rg3-LPs with the control group, it was proved that Blank-MNs, Rg3-MNs and Rg3-LPs did not inhibit cell growth and had good biocompatibility (Figure 3F).

#### Dissolution of Rg3-MNs *in vivo*

Dissolvable MNs have the property of dissolving after being inserted into the skin and releasing medicines. Rg3-MNs have a complete shape before insertion, and the needle tip is sharp. After 5 min of insertion, the needle tip dissolved and became blunt. Within 30 min, the MNs were almost completely dissolved (Figure 3G). This indicates that after the prepared MNs enter the skin, the drug is dissolved and released by absorbing the tissue fluid, subsequently, the medication is transported to the dermal region housing the HFs.

### Biodistribution of Rg3-MNs

#### Penetration depth study

**Figure 4.**
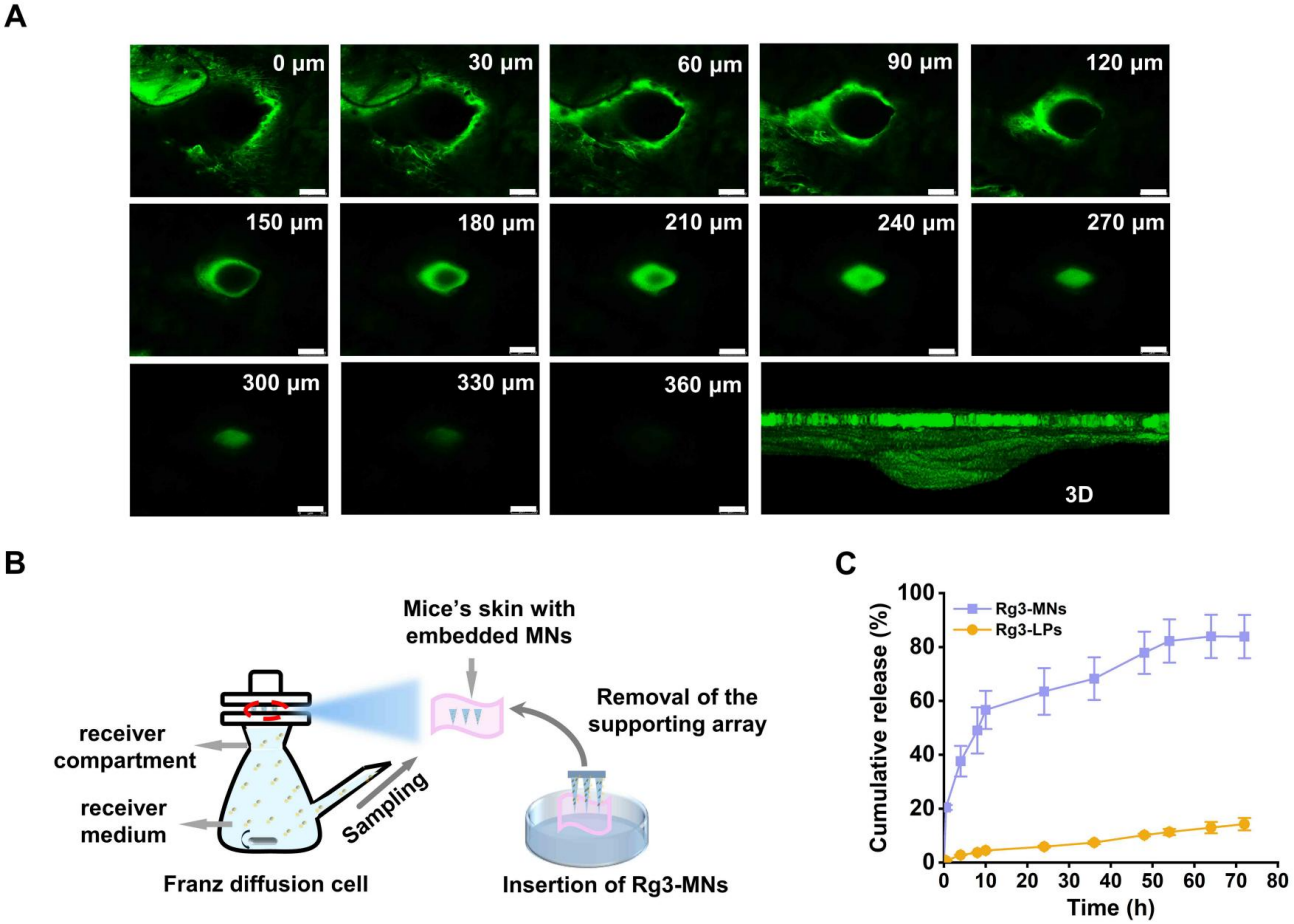
Skin penetration characterization and drug release of the Rg3-MNs. (**A**) CLSM images of C57BL/6 mice skin (scale: 100 μm). (**B**) Schematic diagram of the transdermal drug delivery research. (**C**) *In vitro* transdermal drug release curve. Data are presented as mean ± SD (*n* = 3).

As shown in Figure 4A, the reconstructed 3D fluorescence image was shown as a triangle, and the depth of FITC green fluorescence entering the skin was about 360 μm, which indicated that Rg3-MNs finally reached the dermis, and the drug could quickly spread. Nonetheless, this depth is shorter than the microneedle's geometrical height (∼900 μm). This discrepancy might be attributed to skin deformation and its viscoelastic properties during the insertion process.

The skin permeability of MNs was evaluated by *in vivo* imaging when topical or MNs-encapsulated IR780 was applied. The strength of the two groups of IR780 increased continuously to 120 min (Supplementary Figure S1A). However, the fluorescence intensity of IR780-MNs could cover the back within 30 min, and the signal intensity generated was higher than that generated by the liposome group, which proved the superior penetration efficiency of MNs.

#### Release study of Rg3-MNs *in vivo*

The DiR-LPs group's *in vivo* fluorescence imaging lasted only one day, but the DiR-MNs group revealed a continuous fluorescence signal after five days (Supplementary Figure S1B). Because the Rg3 liposome was released from the HA/BSP needle material as a result of the liposome sustained-release medication action, the MNs group demonstrated a significant fluorescence effect after 1 day of therapy.

#### Transdermal drug release *in vitro*

Figure 4C shows the *in vitro* transdermal release curve of the drug. After 72 h, the cumulative release of Rg3-LPs was only 14.27%. The results illustrated that LPs had a certain ability for transdermal drug delivery, but the efficiency was low. MNs created micron-sized channels to deliver drugs by inserting the SC, consequently, Rg3-LPs showed a good release trend. The cumulative release reached 56.68% within 10 h, and the release trend slowed down after that, but after 72 h, the cumulative release amount reached 83.87%.

#### Evaluation of the therapeutic effect of promoting hair growth *in vivo*

**Figure 5.**
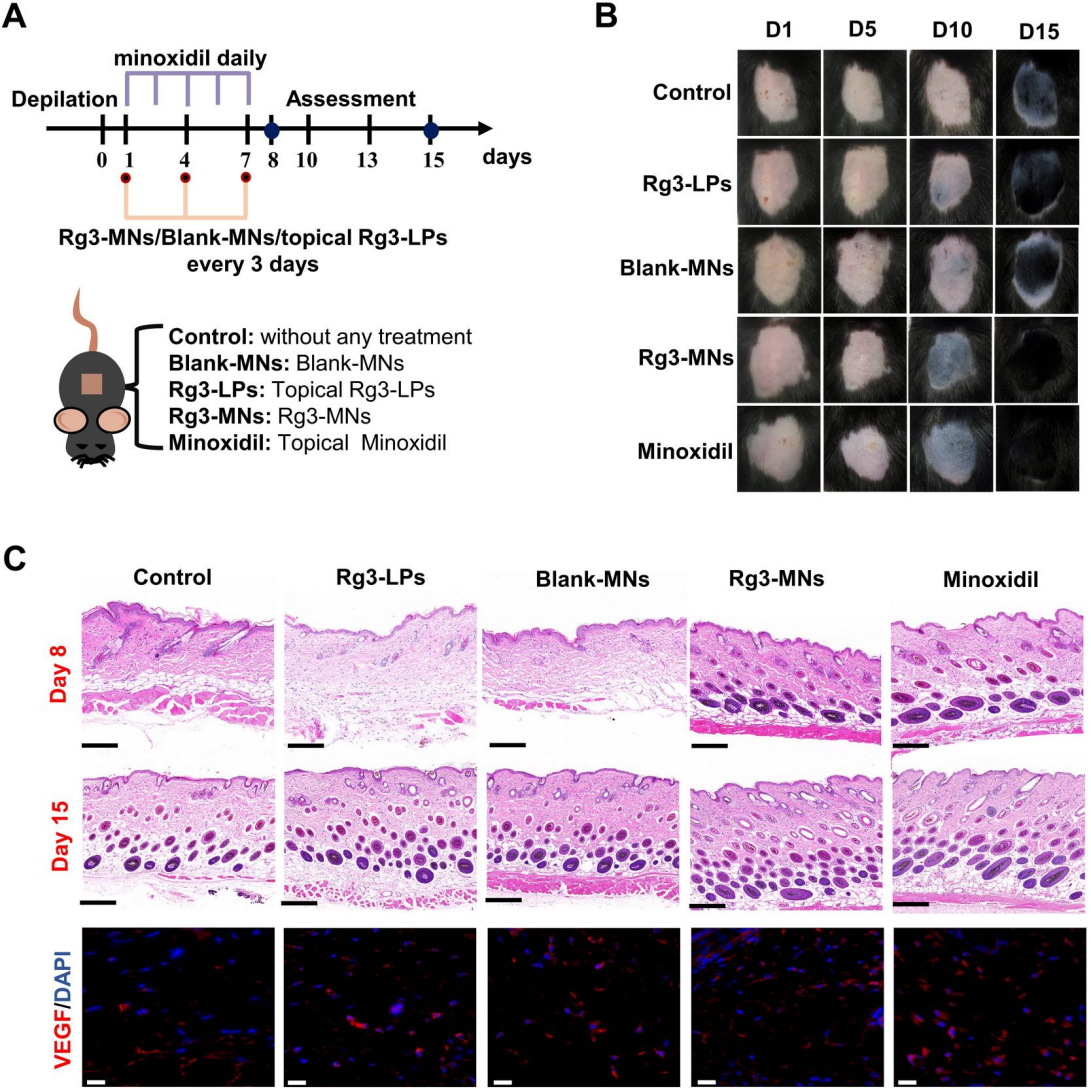
Evaluation of promoting hair growth in telogen mice. (**A**) Treatment of telogen mice model. (**B**) Images depicting the hair regrowth status of each group. (**C**) H&E staining on day 8 and 15 post-depilation. Scale bar: 200 μm. Representative micrographs of VEGF staining of depilated skin on day 8 post-depilation. Scale bar: 100 μm.

**Figure 6.**
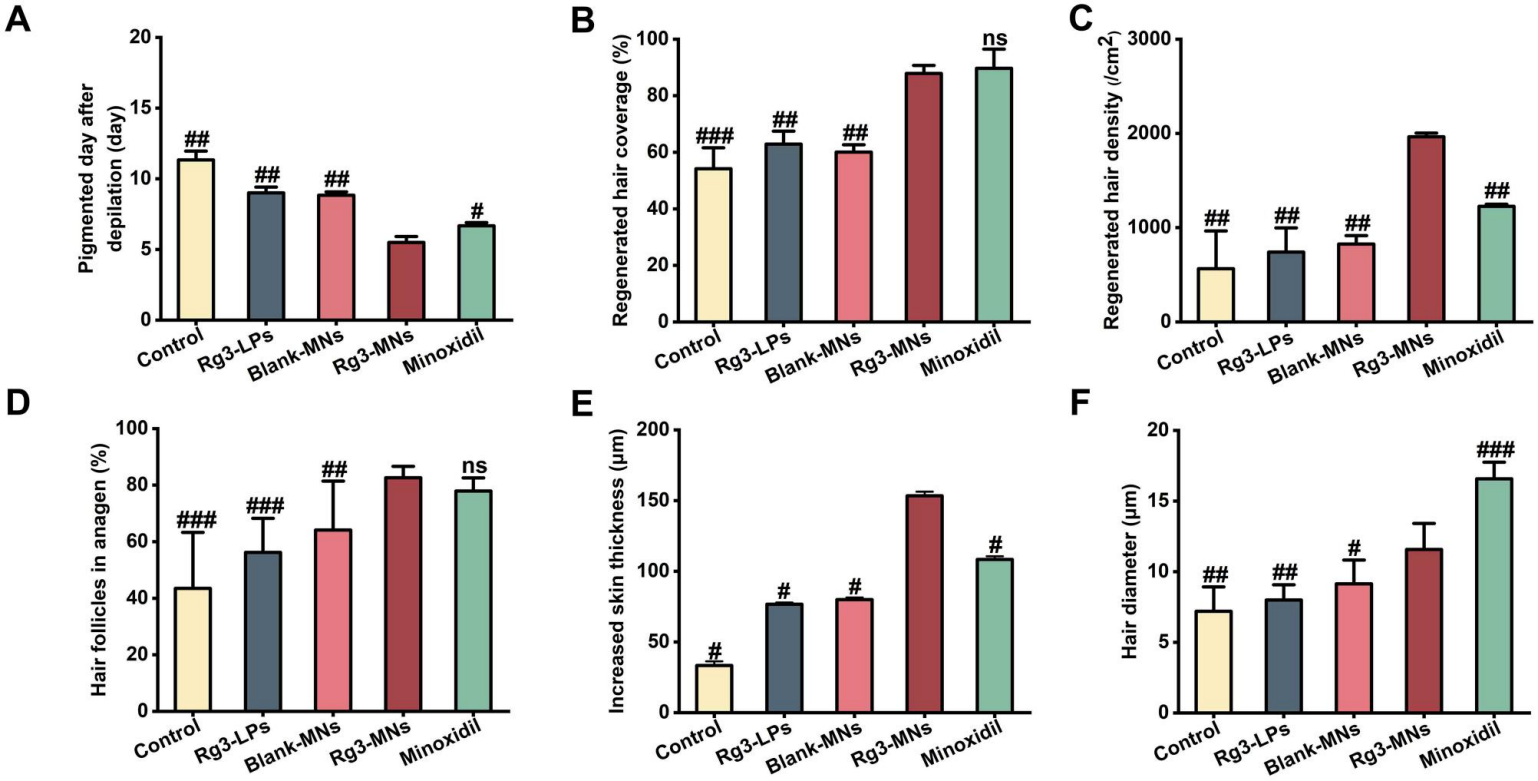
Evaluation of promoting hair growth in telogen mice. (**A**) Pigmented day after depilation. (**B**) Regenerated hair coverage. (**C**) Regenerated hair density. (**D**) Hair follicles in anagen. (**E**) Increased skin thickness. (**F**) Hair diameter. Bars represent mean ± SD (*n* = 3). ns, nonsignificant (*P *>* *0.05), ^#^*P *<* *0.05, ^##^*P *<* *0.01, ^###^*P *<* *0.001 vs the Rg3-MNs group.

To investigate the effect of each group of drugs on hair growth, the C57BL/6 mouse model with HFs in the telogen phase is selected. It is worth noting that the color of the dorsal skin of C57BL/6 mice can directly reflect the growth of HFs. When the HF is in the telogen phase, the skin is fleshing pink, and when the HFs begin to activate the transition to the growth phase, the skin color changes accordingly to light gray until it deepens to dark brown or black. Therefore, the change of color on the back of mice during the experiment can visually compare the treatment of each group of mice. In Figure 5B, before treatment, the skin hue of all groups appeared pink, suggestive of the telogen phase in mouse HFs. After various treatments, the skin color of mice in the Rg3-MNs group became black first, expressing that Rg3-MNs accelerated the induction of HFs from the telogen to the anagen and reached hair coverage similar to minoxidil after 15 days, and the efficacy was significantly better than other groups (Figure 6A, B and D). The diameter of regenerated hair in the Rg3-MNs group could also achieve a similar effect as minoxidil, but because HA had the effect of increasing hair density, the effect of regenerated hair density in the Rg3-MNs group was better than that of minoxidil (Figure 6C and F) [21].

To further verify the therapeutic effect of Rg3-MNs on telogen HFs, H&E sections and VEGF immunofluorescence results presented that the Rg3-MNs group could activate HFs and accelerate the growth of HFs. The skin thickness in the Rg3-MNs group exhibited a significantly greater increase compared to the other groups, owing to the change of skin thickness increased significantly during the transition from HFs to anagen (Figures 5C and 6E).

It was worth noting that Rg3-LPs still had a certain penetration ability, which could promote hair growth so the mice in the experimental group still showed hair growth effect after 15 days. The mechanical stimulation caused by Blank-MNs on the skin could enhance the expression of pro-angiogenic factors and induced hair regeneration, while HA could increase hair density, so that the Blank-MNs group showed a certain germinal effect, and the density of regenerated hair was slightly higher than that of Rg3-LPs.

#### Efficacy evaluation of Rg3-MNs on AGA treatment

**Figure 7.**
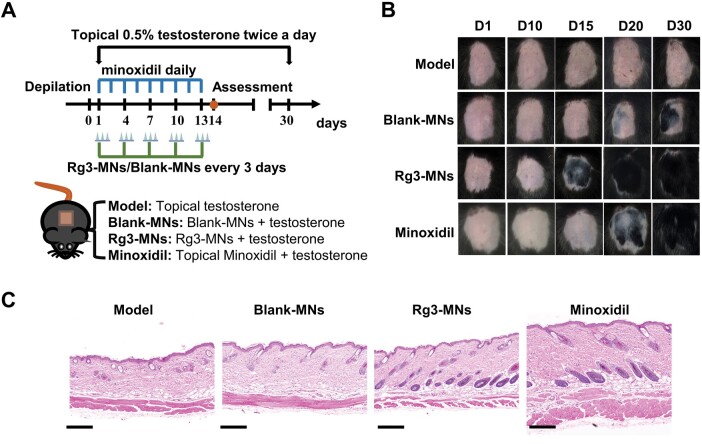
Assessment of hair regrowth in AGA mice *in vivo*. (**A**) The establishment of the AGA mouse model through testosterone solution and the therapeutic approaches implemented differently in the established model. (**B**) Illustrative images showcasing the hair regrowth status in mice from the model, Blank-MNs, Rg3-MNs and minoxidil groups. (**C**) H&E staining of the treated skin at day 14 post-depilation. Scale bar: 200 μm.

**Figure 8.**
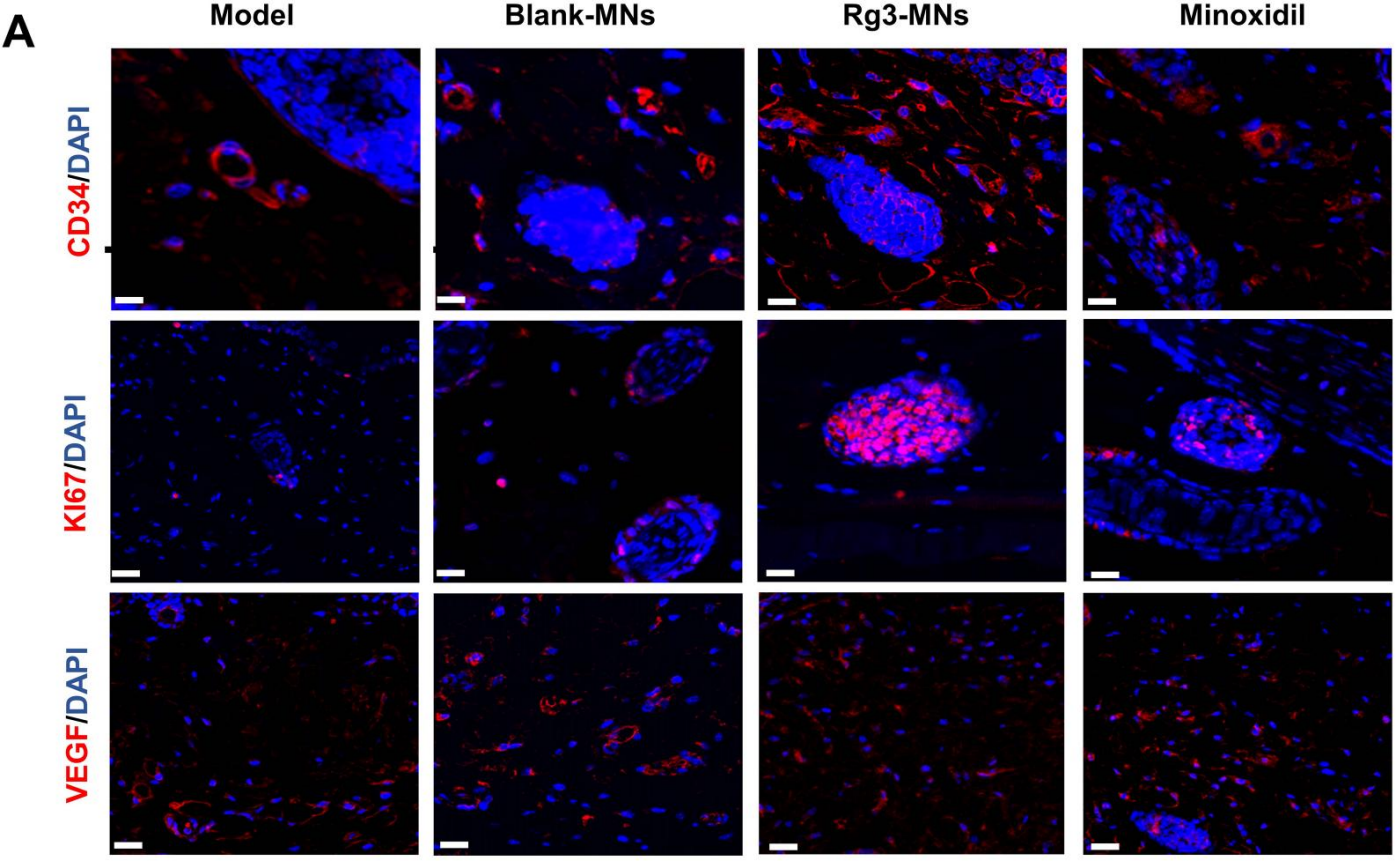
Evaluation of Rg3-MNs system for hair regeneration. (**A**) Representative micrographs of CD34, KI67 and VEGF staining of depilated skin on day 14 post-depilation. Scale bar: 100 μm.

**Figure 9.**
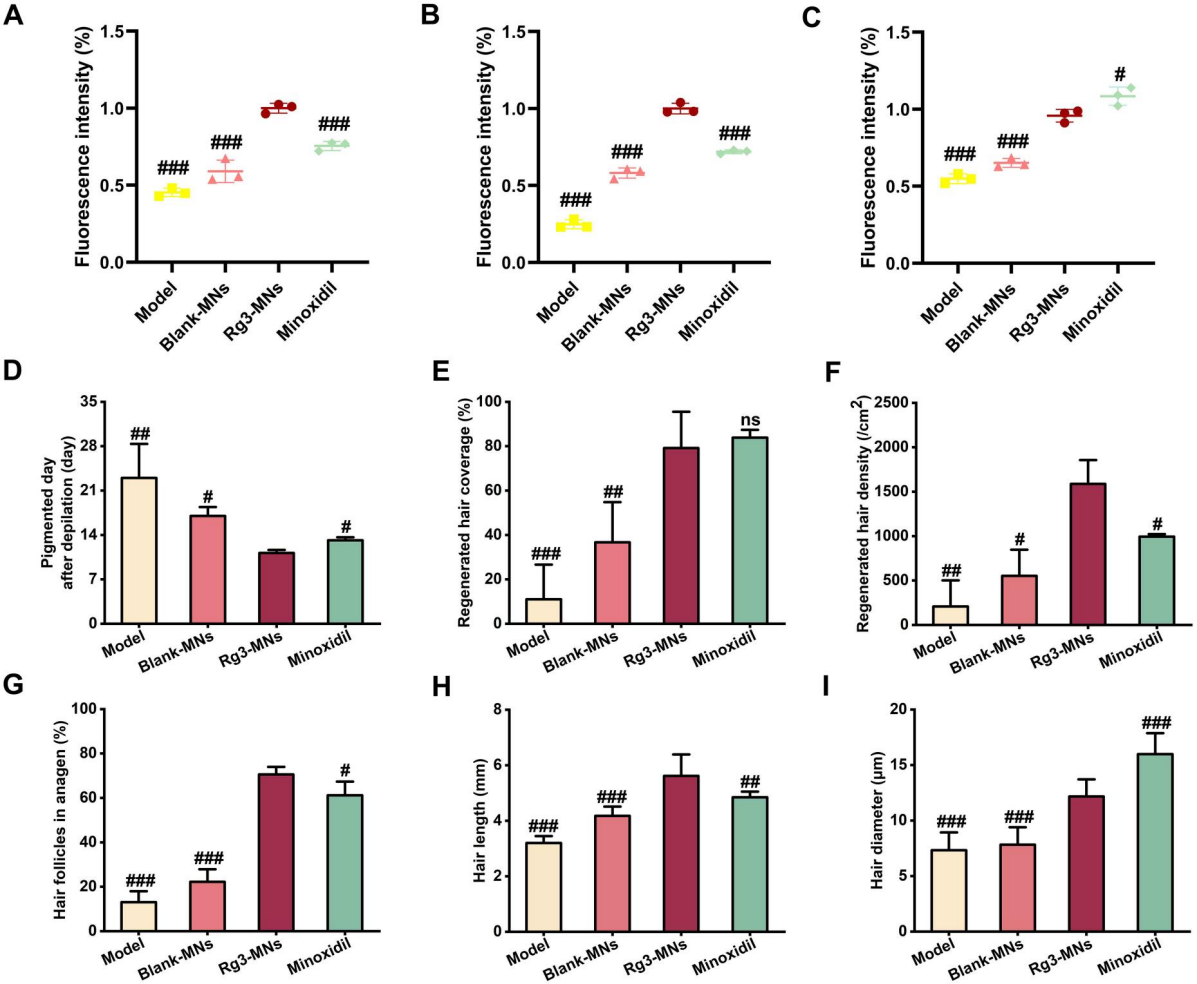
Assessment of hair regrowth in AGA mice *in vivo*. (**A**) Relative CD34 expression. (**B**) Relative Ki67 expression. (**C**) Relative VEGF expression. (**D**) Pigmented day after depilation. (**E**) Regenerated hair coverage. (**F**) Regenerated hair density. (**G**) Hair follicles in anagen. (**H**) Hair length. (**I**) Hair diameter. Scatter plots represent mean ± SD (*n* = 3). ns, nonsignificant (p > 0.05), ^#^*P *<* *0.05,^##^*P<0.01,*^###^*P *<* *0.001 vs the Rg3-MNs group.

To delve deeper into the hair regeneration capabilities of Rg3-MNs *in vivo*, we established the AGA mouse model. During the experimental period, most of the depilated mice in the model group were always pink, with less regenerated hair, and the coverage rate of regenerated hair was much lower than that of other groups. While the Blank-MNs, Rg3-MNs and minoxidil groups could complete the HFs activation within 20 days, and demonstrated diverse hair coverage after 30 days, which proved that the testosterone-induced AGA model was successfully established (Figures 7B and 9D–E). It was noted that the HFs in the Rg3-MNs group began to transform, the skin color became black first and the length of regenerated hair was the longest (Figure 9H). With a low administration frequency, the regenerated hair rate similar to that of minoxidil could be achieved, and the density of regenerated hair increased, which verified that Rg3-MNs had a better therapeutic effect on AGA (Figure 9F and I). The H&E section on the 14th day and the proportion of HFs in the anagen phase verified that the HFs of the Rg3-MNs group reached the anagen phase first (Figures 7C and 9G). Additionally, in Figures 8A and 9A–C, the expression of CD34 and Ki67 in the Rg3-MNs group was the highest, while the expression of CD34, Ki67 and VEGF in the model group was the lowest, again indicating that testosterone inhibited hair growth. This indicates that Rg3-MNs can enhance the expression of VEGF and CD34, promote angiogenesis around HFs, accelerate hair regeneration and treat AGA.

#### Rg3-MNs elevated mRNA expression of Wnt/β-catenin signaling pathway

The sequence changes of RNA before and after Rg3-MNs treatment of testosterone-induced AGA model were analyzed. Analysis revealed that following Rg3-MNs treatment, 823 genes were up-regulated and 644 genes were downregulated. Among these genes, we identified genes related to the HF growth stage, which are Wnt3a and Wnt10b (Supplementary Figure S2A–D). The high expression of Wnt3a and Wnt10b significantly promoted the formation of HF and hair growth induced by dermal papilla [44, 45]. To identify the functionality of differentially expressed genes, Go and KEGG enrichment analysis were used for annotation, and it was found that Rg3-MNs treatment caused significant enrichment of Wnt/β-catenin pathway related to HF growth.

#### Rg3-MNs activated the Wnt/β-catenin signaling pathway

The Rg3-MNs group had significantly increased expression of Wnt10 b and Wnt3 a than the model group, and these proteins were linked to the activation of the hair cycle (Supplementary Figure S2E–G). The control group was not administered testosterone to prevent hair development, and hair-related proteins were activated. However, WB results showed that Rg3-MNs could still dramatically raise the expression level of Wnt10 b, confirming that Rg3-MNs can enhance hair growth in mice through the Wnt/β-catenin signaling pathway.

## Discussion

The number of persons experiencing hair loss is increasing, and there is a younger tendency. Although minoxidil and finasteride are widely employed to treat alopecia, their clinical efficacy remains restricted because of their side effects and the barrier effect of SC [46]. MNs combine the advantages of percutaneous and subcutaneous injection to form a unique drug delivery technology capable of effectively delivering pharmaceuticals by physical puncture of SC regardless of molecular weight. When MNs are utilized as drug delivery vehicles, they can up-regulate VEGF by mechanical stimulation, promote angiogenesis for hair growth and improve the density and quality of new hair [25, 43]. Therefore, in this study, we designed a soluble MN patch containing cholesterol-free Rg3 liposomes for alopecia treatment. The MNs were 900 μm high, and needle spacing was 700 μm, which can effectively reduce the insertion force [47]. MNs consist of biodegradable and hydrophilic materials (i.e. HA and BSP) [48]. HA can also improve the environment for the proliferation, migration and aggregation of human dermal papilla cells (HDPCs), resulting in hair regeneration and increased skin thickness [49]. Compared with roller MNs combined with minoxidil nanoparticles and valproic acid composite paste system for the treatment of AGA, the Rg3 dissolved MNs prepared in this study are convenient to use, simple and safe in raw materials, and a have good therapeutic effect [50]. Among them, cholesterol-free Rg3 liposomes avoid excessive cholesterol intake and affect liver and gallbladder metabolism, while also avoiding cholesterol as a precursor of testosterone biosynthesis, that generates DHT, which inhibits hair regeneration [51]. Rg3-MNs were inserted into the skin and dissolved in 30 min to achieve Rg3-LPs transdermal delivery while avoiding the influence of the SC barrier on liposome absorption. Furthermore, the mechanical robustness of Rg3-MNs allowed them to effectively penetrate mouse skin, where HF cells reside, and continually release Rg3-LPs for more than 5 days. It is worth mentioning that Rg3-MNs show significant hair regrowth in both TE and AGA models when compared to the blank and model groups. Furthermore, Rg3-MNs had a similar hair regeneration efficacy to minoxidil with required less frequent administration and produced higher-quality regenerated hair. In skin HF tissue, Wnt signaling regulates the growth cycle of hair papilla cells. When Wnt signal transduction is inhibited, HFs enter the involution stage prematurely, resulting in alopecia [52]. As a result, activating Wnt signaling can accelerate HFs to begin development and promote new hair creation. Interestingly, we observed that Rg3-MNs could up-regulate the expression of Wnt10b and Wnt3a through transcriptome and Western blotting analysis, suggesting that Rg3-MNs may promote local hair regeneration by activating the Wnt/β-catenin signaling pathway.

This study provides a new treatment for hair regeneration, although several limitations exist, such as limited drug loading in MNs and inadequate drug targeting. Although the drug dose of each MN patch is approximately 130 μg, it has a therapeutic impact on mice in the experiment, but it may not be sufficient for human application. Though Rg3-LPs avoid the barrier effect of SC, they reduce medication efficacy due to inadequate targeting of HFs. As a result, future research will focus on modifying Rg3-LPs to target HFs. Others, we can consider the combination of Rg3 and aminolevulinic acid (ALA) and use photodynamic therapy to explore the therapeutic potential of photosensitizers in the area of alopecia [53]. It is also a future research direction to improve the manufacturing method of MNs and prepare core–shell dissolvable MNs to encapsulate more drugs and release them rapidly [54].

## Conclusion

Using ginsenoside Rg3 as a membrane material, we designed a cholesterol-free liposome in this study to prevent cholesterol from acting as a precursor to testosterone production, which might then be transformed into DHT, which could harm HFs and inhibit hair regeneration. Rg3-LPs was successfully loaded into MNs based on HA/BSP. The Rg3-MNs patch had sufficient mechanical strength for skin penetration and mechanical stimulation no skin irritation during use and had good biocompatibility. It could be quickly dissolved and release drugs. The microporous channels formed after the insertion of the Rg3-MNs patch significantly promoted the delivery of Rg3-LPs to the HFs region. Compared with the daily application of minoxidil, Rg3-MNs up-regulated the expression of CD34 and Ki67, and promoted the expression of Wnt3a and Wnt10b genes, activating the Wnt/β-catenin pathway, accelerating HFs transformation into anagen, promoting hair regeneration, and enhanced the quality of regenerated hair at a lower frequency of administration. Rg3-MNs demonstrated better therapeutic effects on the TE model and AGA model.

## Supplementary Material

rbae086_Supplementary_Data

## Data Availability

Data will be made available on request.
