# Supplementary material for: Dissolvable microneedles loaded ginsenoside Rg3 liposome: a transdermal delivery approach for alopecia treatment
Source: Regen Biomater. 2024 Jul 16;11:rbae086. doi: 10.1093/rb/rbae086 (PMC11333571; doi:10.1093/rb/rbae086)
Supplement: rbae086_Supplementary_Data [file rbae086_supplementary_data.docx]

**Supporting Information**

**Dissolvable Microneedles Loaded Ginsenoside Rg3 liposome: A Transdermal Delivery Approach for Alopecia Treatment**

Qin Yang ^a^, Peng Guo ^a^, Pengkun Lei ^a^, Qiaolin Yang ^a^, Yuchun Liu ^a^, Ya Tian ^a^, Wen Shi ^a^, Chunxiao Zhu ^a^, Min Lei ^d^, Rui Zeng ^b, c, *^, Chen Zhang ^a, *^, Yan Qu ^a, *^

*^a^ State Key Laboratory of Southwestern Chinese Medicine Resources, School of Pharmacy, Chengdu University of Traditional Chinese Medicine, Chengdu 611137, China*

*^b^ College of Pharmacy, Southwest Minzu University, Chengdu 610041, China*

*^c^ Key Laboratory of Research and Application of Ethnic Medicine Processing and Preparation on the Qinghai Tibet Plateau*

*^d^ Lu Huo Snow area E Se Limited Liability Company, Chengdu 626500*

**Corresponding authors. E-mail addresses:* [*quyan028@126.com*](mailto:quyan028@126.com) *(Y. Qu),* [*chenzhang_1990@126.com*](mailto:chenzhang_1990@126.com) *(C. Zhang),* [*rzeng@swun.edu.cn*](mailto:rzeng@swun.edu.cn) *(R. Zeng)*

# Biodistribution of Rg3-MNs

## Penetration research in vivo

IR780 was encapsulated in Rg3-LPs to evaluate the penetration efficiency of drugs in different formulations in mice. The fluorescence signal was captured using the IVIS Spectrum imaging platform (Perkin-Elmer) at intervals of 30, 60, 90, and 120 min.

## Release study in vivo

DiR-LPs and DiR-MNs were used to track the release of Rg3-LPs and Rg3-MNs. On days 0, 1, 3, and 5, photographs of mice were captured by IVIS Spectrum (Perkin-Elmer) for in vivo fluorescence imaging.

# RNA-sequencing analysis

RNA purification, reverse transcription, library preparation, and sequencing were conducted by Shanghai Majorbio Bio-pharm Biotechnology Co., Ltd. (Shanghai, China). Adhering to the guidelines provided by the manufacturer, total RNA was extracted from the tissue using QIAzolLysisReagent (Qiagen, Germany). Subsequently, the quality of RNA was assessed utilizing the 5300 Bioanalyser (Agilent), while its quantity was measured with the ND-2000 (NanoDropTechnologies). Then, double-stranded cDNA was generated utilizing the SuperScript cDNA synthesis kit from Invitrogen, California, employing random hexamer primers supplied by Illumina. Subsequently, the cDNA underwent end-repair, phosphorylation, and the addition of an ‘A’ base, adhering to Illumina's library construction protocol. Followed by PCR amplified using Phusion DNA polymerase (NEB), after quantified by Qubit 4.0, paired-end RNA-seq sequencing library was sequenced with the NovaSeq Xplus sequencer (2 ×150 bp read length).

To demonstrate the similarities and disparities between the two groups, DEseq2 software package was used to standardize the RNA-Seq data and cluster analysis was performed. RSEM software was used to construct volcanic visualization plots and differential gene Venn plots between different objects, and a differential expressed gene (DEG) thermal map was used. GO and KEGG enrichment were conducted utilizing GOseq and KOBAS.

# Western blot analysis

After 14 days of administration, the mice were euthanized, and the dorsal skin tissue was excised. Proteins were isolated from the skin and transferred to PVDF membranes. The membrane was blocked and diluted with appropriate primary antibody targeting Wnt3a (1:1000; CST), Wnt10b (1:1000 dilution; Affinity), and β-catenin (1:1000 dilution; Servicebio) at 4 °C. Then, they were diluted with anti-rabbit IgG / HRP (1:1000 dilution; Mult Sciences) incubated and captured by a chemiluminescence substrate (ChemiScope). Utilizing Image J software, the grayscale intensity of protein bands was quantitatively analyzed.

**
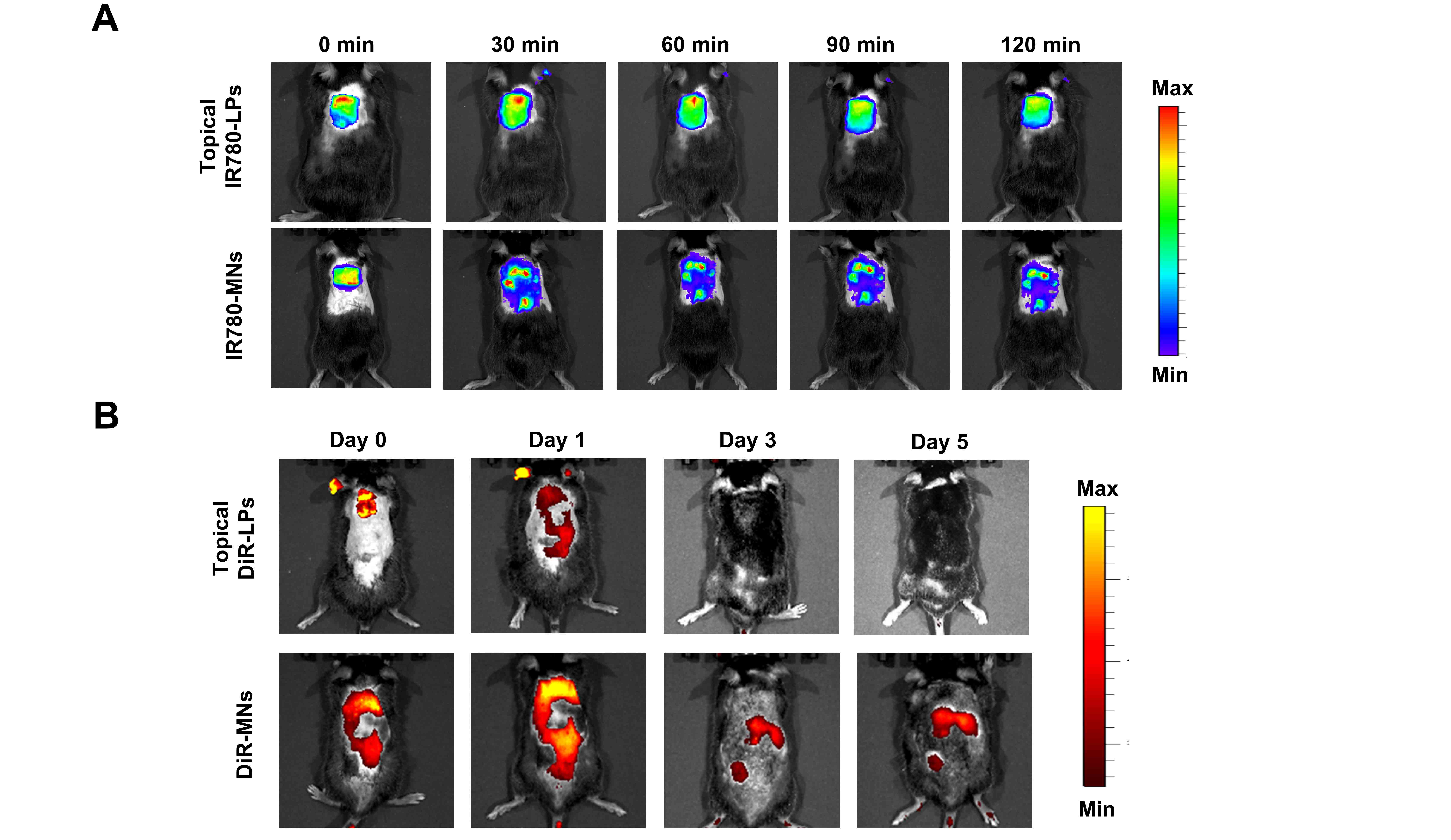
**

**Figure S1**. Skin penetration characterization of the Rg3-MNs. (A) CLSM images of skin permeability efficiency of IR780-LPs and IR780-MNs. (B) CLSM images of DiR-LPs and DiR-MNs retained.


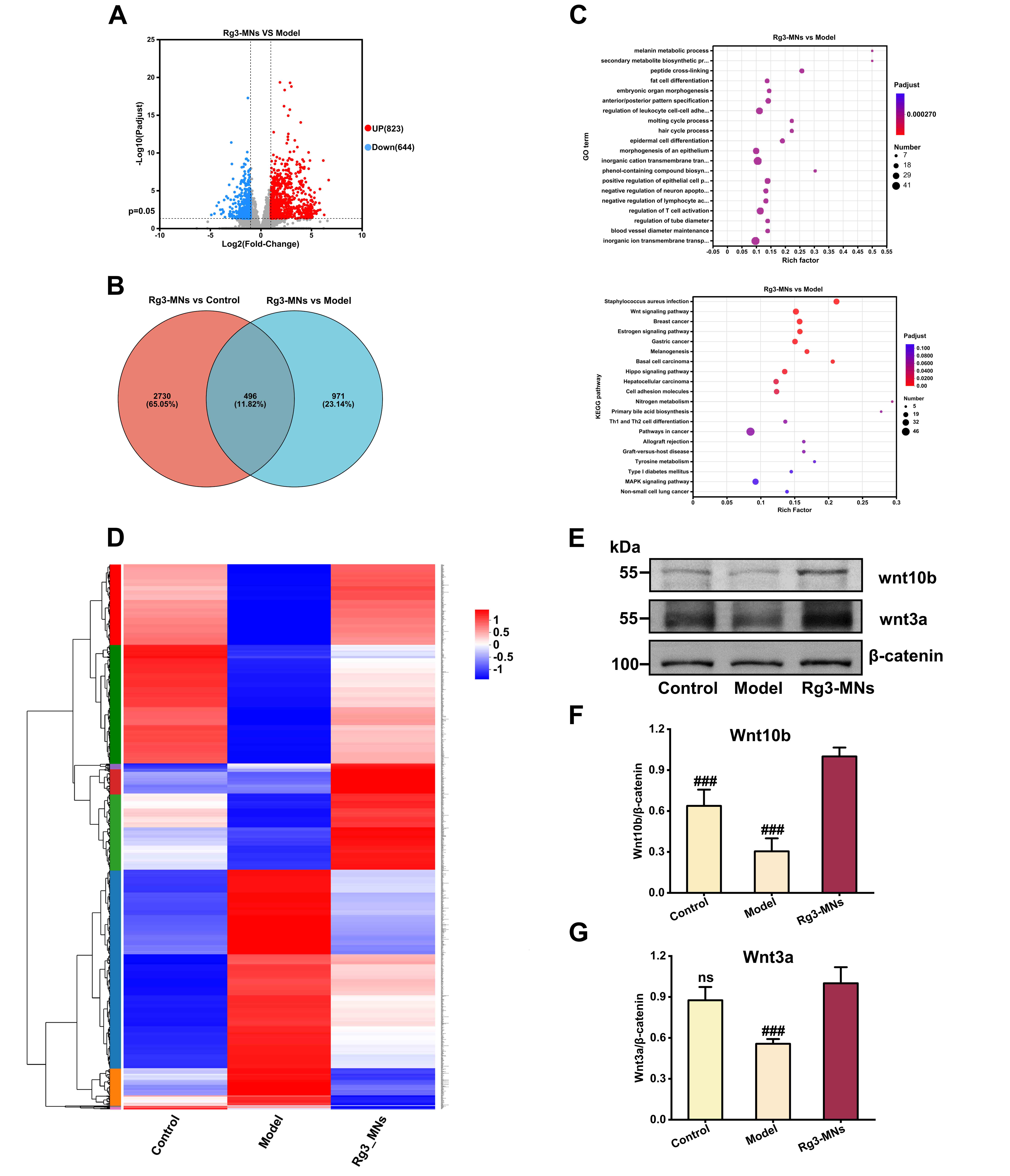


**Figure S2**. Transcriptome sequencing analysis and Rg3-MNs triggered the Wnt/β-catenin signaling pathway. (A) Sequencing analysis of AGA model treated with Rg3-MNs. Volcano plot revealed that 823 genes were unregulated and 644 genes were downregulated in the AGA model after Rg3-MNs treatment. (B) Venn diagram illustrated DEGs comparing Rg3-MNs with Control and Rg3-MNs with Model. (C) Analysis of Go term and KEGG pathway of regulatory genes after Rg3-MNs treatment of AGA. Color represents *P* value. (D) Categorical heatmaps depicting the significantly altered genes across three groups (n=4). (E-G) Western blot analysis of β-catenin, Wnt10b(F), Wnt3a(G) for the back skin samples after 14 days. Bars represent mean ± SD (n=5), ns, nonsignificant (*p* > 0.05), ^###^*p* < 0.001 vs the Rg3-MNs group.
